# Treatment of Open Pediatric Tibial Fractures by External Fixation Versus Flexible Intramedullary Nailing: A Comparative Study

**DOI:** 10.5812/atr.13826

**Published:** 2013-12-01

**Authors:** Hossein Aslani, Ali Tabrizi, Ali Sadighi, Ahmad Reza Mirblok

**Affiliations:** 1 Pediatric Orthopedic, Tehran University of Medical Sciences,Tehran, IR Iran; 2 Orthopedic Surgery, Shohada Educational Hospital, Tabriz University of Medical Sciences, Tabriz, IR Iran; 3 Shohada Educational Hospital, Tabriz University of Medical Sciences, Tabriz, IR Iran; 4 Orthopedic Surgery, Poursina Educational Hospital, Guilan University of Medical Sciences, Rasht, IR Iran

**Keywords:** Fractures, Open, External Fixator, Fracture Fixation, Child

## Abstract

**Background:**

Tibial fractures are the third most common pediatric long-bone fracture after forearm and femoral fractures. Approximately 50% of pediatric tibial fractures occur in the distal third of the tibia. This is followed by midshaft tibial fractures (39%), and least commonly, the proximal third of the tibia is involved. Tibial fractures in the skeletally immature patient can usually be treated without surgery but tibial fractures resulting from high energy traumas are of special importance considering type of the selected treatment method affecting the children future. Manipulation and casting are regarded as definite treatments for children tibial fractures. They are used following compartment syndrome in poly-trauma, neurovascular damages, open fractures, and fasciotomy cases.

**Objectives:**

In children, most open fractures occur due to high energy traumas and inappropriate treatment of the fractures may result in several complications. Flexible intramedullary nailing is one of the popular options as an effective method of treating long-bone fractures in children. The external fixator is used in cases with severe injuries and open fractures. The present study aims at comparing results of these two treatment methods in the open pediatric tibial fractures.

**Materials and Methods:**

In this descriptive analytical study, 32 patients with open tibial fractures were treated with either fixator (n = 18) or TEN nails (n=14) during 2006-2011. Some patients were treated with a combination method of TEN and pin. The results were evaluated considering infection, union, mal-union, and re-fracture and the patients were followed up for two years.

**Results:**

Mean time required for fracture union was 12.5 (11-14) and 11.8 (10-12) weeks for the external fixator and TEN groups, respectively. There was no statistical difference in time of union between the two methods. The main complications in external fixation were infection around the pin 4 (22.2%), leg-length discrepancy 2 (11.1%) and re-fracture 4 (22.2%). In the TEN group, 2 cases (14.2%) of painful bursitis were observed at the entry point of TEN and the pin was removed earlier. There was not any report of mal-union requiring correction in the groups. No complication was seen in 6 patients treated with a combined method of pin and flexible intramedullary nails.

**Conclusions:**

Although external fixation in open pediatric fractures and severe injuries is recommended, intramedullary nailing is also an effective method with low complications. Combining pins and flexible intramedullary nails is effective in developing more stability and is not associated with more complications.

## 1. Background

Tibial fractures are the third most common pediatric long-bone fracture after forearm and femoral fractures (1). Approximately 50% of pediatric tibial fractures occur in the distal third of the tibia (1). This is followed by midshaft tibial fractures (39%), and least commonly, the proximal third of the tibia is involved (1). Tibial fractures in the skeletally immature patient can usually be treated without surgery but tibial fractures resulting from high energy traumas are of special importance considering type of the selected treatment method affecting the children future (2, 3). Manipulation and casting are regarded as definite treatments for children tibial fractures (4). They are used following compartment syndrome in polytrauma, neurovascular damages, open fractures, and fasciotomy cases (4). Flexible intramedullary nails (FIN) have been used increasingly since the 1980s for the management of paediatric tibial and femoral fractures (4). Short-term immobilization, returning joints range of motion, lack of any stiff joint, short-term hospitalization, and low costs are regarded as advantages of the flexible nails. According to study by Pandya et al. immediate flexible nailing of open pediatric tibial shaft fractures can be safely performed with minimal risk of wound or infectious complications (5). Prolonged bone healing (particularly in Gustilo type 2 or 3 injuries) should be expected in patients who undergo immediate flexible nailing of their open fractures (5). External fixators are used in open complex fractures resulting from high energy traumas as well as cases of several damages (4). However, they are associated with some complications including pin tract infection and scar where the pins are located (4, 6). There are few studies comparing the results of these two surgical methods in grade III open tibial fractures of children. Therefore, it was tried to compare the results of the two-mentioned methods.

## 2. Objectives

In children, most open fractures occur due to high energy traumas and inappropriate treatment of the fractures may result in several complications. Flexible intramedullary nailing is one of the popular options as an effective method of treating long-bone fractures in children. The external fixator is used in cases with severe injuries and open fractures. The present study aims at comparing results of these two treatment methods in the open pediatric tibial fractures.

## 3. Materials and Methods

This retrospective descriptive-analytical study was conducted at Trauma Center in Northwestern Iran (Shohada hospital affiliated by Tabriz University of Medical Sciences) during 2006-2011. In this study, 32 children (younger than 14 years) suffering from Gustilo grade A and B III open fracture of tibia were admitted at the emergency department of the center and evaluated. 

The patients were followed-up at least for two years. Children with Gustilo grade III A & B tibial shaft open fractures were selected. Children with history of lower extremities fractures, systemic and metabolic diseases, and skeletal congenital diseases were excluded. The fractures often resulted from high energy motor vehicle accidents. The children were matched considering age, gender, damage mechanism, and open fracture type (grade III) and associated damages as well as neurovascular complications were recorded for all patients.

**Figure 1.**
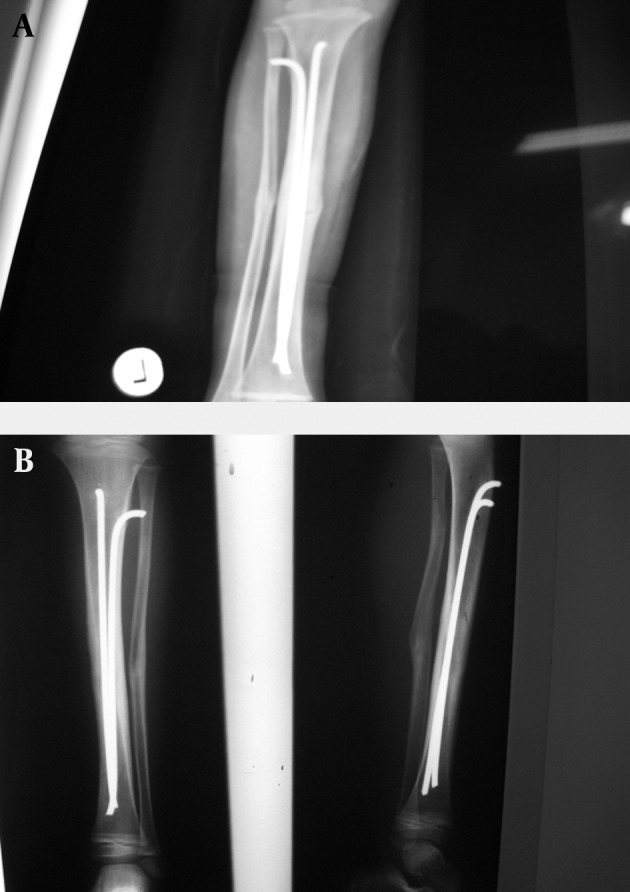
Fixation of Tibia by Flexible Intramedullary Nail.

**Figure 2.**
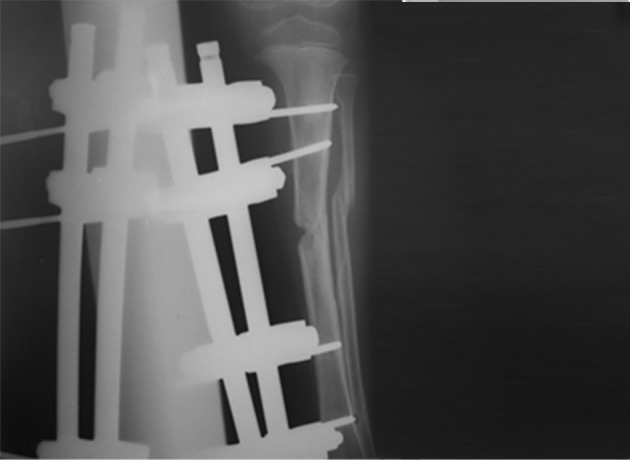
Fixation with Combination of Pin and Flexible Intramedullary Nail.

**Figure 3.**
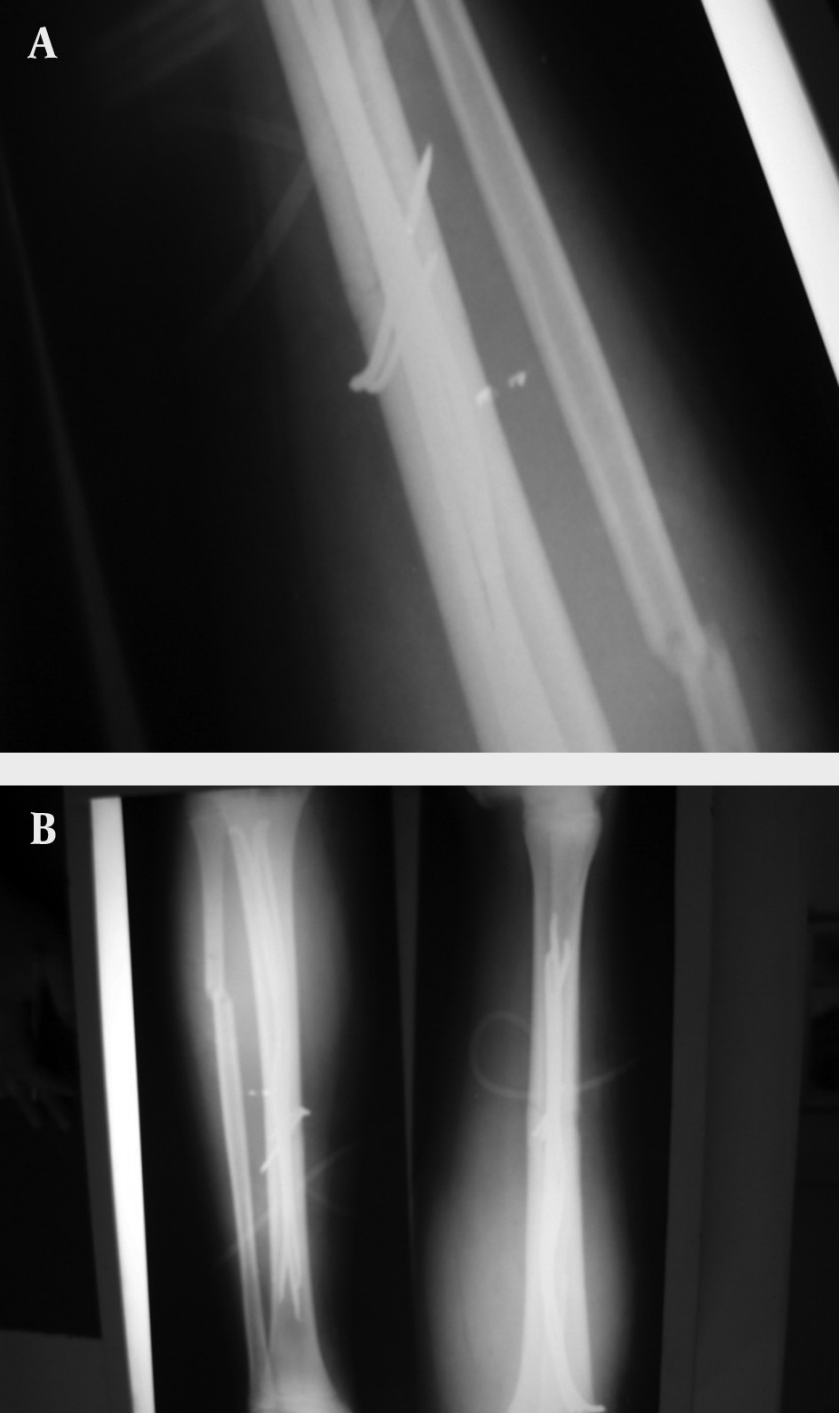
Fixation with External Fixator in Open Tibial Fracture.

While the children were admitted at the emergency department of the center, they underwent prophylaxis using first generation antibiotic of cephalosporins (cepharolin 100mg/kg/day) and gentamicin. In severe cases, third antibiotic (penicillin Crystal) were added to the treatment regime, if required. All patients underwent washing and primary debridement operation within the first 6 hours of admission at the emergency department. According to the attending surgeon, the patients were treated either using external fixator or intramedullar nails during the first day of hospitalization. Union of the fracture was controlled through clinical examinations such as lack of pain, tenderness, crepitation at the fractured area as well as using radiography of both lateral and anteroposterior (AP) views during the follow-up period. Delayed union was regarded as non-union for more than 6 months. When intramedullar nails were inserted, surgical treatment was controlled through fluoroscopy. In some cases, pins were used to fix the fractured area (Figures 1 and 2). Unilateral monotube system was used to stable the fracture in external fixation method (Figure 3). Eligible patients who provided informed consent were included in the study. An Ethics Committee of Tabriz University of Medical Sciences approved the study and the study conforms to the ethical principles contained in the declaration of Helsinki. 

All statistical analyses were performed using SPSS 16. The Chi- square and Fisher's exact tests were used for qualitative variables; the independent T-test was applied for quantitative variables. In this study, P < 0.05 was regarded meaningful.

## 4. Results

In this study, 32 children with tibial open fracture treated with the external fixation method (n = 18) and flexible intramedullar nails (n = 14) were compared. Pins were used to increase stability of the fractured area in 6 patients (42.8%) treated with flexible intramedullar nails. Demographic findings of the understudy children and the associated damages are shown in Table 1. 

**Table 1.** Comparing Demographic Findings and Other Associated Complications Between two Groups Treated with External Fixator and Intramedullar Nails

Variable	External Fixator Group (n = 18)	Flexible Nails Group (n = 14)
**Age, y**	10.5 ± 3.2	11 ± 3.7
**Female/Male, No. (%)**	10/8 (55.5/44.5)	8/6 (57.1/42.9)
**Head closed damage, No. (%)**	3 (16.6)	1 (7.1)
**Thorax and abdomen damage, No. (%)**	2 (11.1)	0 (0.0)
**Pelvic fracture, No. (%)**	1 (5.5)	0 (0.0)

Follow-up results have been summarized in Table 2. There was not any meaningful difference between two groups considering deep infection of the fractured area and osteomyelitis was not observed in any group. 

**Table 2.** Comparing Complications Between two Treatment Methods of External Fixator and Intramedullar Nails

Variable	External Fixator Group (n = 18)	Flexible Nails Group (n = 14)
**Mean time of union, w**	12.5 ± 1.4	11.8 ± 1.2
**Infection surrounding pins, No. (%)**	4 (22.2)	0 (0.0)
**Painful bursitis, No. (%)**	0 (0.0)	2 (14.2)
**Sagittal plane angulation ( > 10° recurvatum), No. (%)**	1 (5.5)	0 (0.0)
**Coronal plane angulation (> 10° varus), No. (%)**	1 (5.5)	0 (0.0)
**Re-fracture, No. (%)**	4 (22.2)	0 (0.0)
**Mal-union, No. (%)**	0 (0.0)	0 (0.0)
**Limb length difference > 1cm, No. (%)**	2 (11.1)	0 (0.0)

Infection surrounding pins created some problems in 4 cases (22.2%) and it was necessary to change place of the pins. In the TEN group, 2 cases (14.2%) of painful bursitis was observed at the entry point of TEN and the pin was removed earlier. There were four cases (22.2%) of tibial re-fracture in the external fixator group. Leg-length discrepancies of between 1.5 cm and 2 cm following external fixator of multifragmentary tibial fractures occurred in 2 (11.1%) patients and were treated by epiphysiodesis of the contralateral leg. There was not any report of mal-union requiring correction in none of the groups. No infection was seen in those patients treated with a combined method of pin and flexible intramedullary nails. No patient demonstrated evidence of growth arrest after intramedullary nail insertion. In our samples were not any compartment syndromes.

## 5. Discussion

Pediatric tibial shaft fractures usually are not complicated and can be treated with reduction and casting (1). Patients with displaced fractures are reduced in the operating room with fluoroscopy to facilitate the reduction (1). Tibial fractures have been treated non-surgically within the last two decades and immobilization using cast was regarded as a standard treatment (1, 7). However, surgical treatment is recommended in cases with several damages, high energy traumas, open fractures, and compartment syndrome (8). Although cast immobilization remains the standard treatment for appropriate fractures of the tibia fixation is particularly beneficial for children who have sustained multiple injuries from high energy trauma. Developing flexible intramedullar nails brought great evolutions in treating children long-bone fractures and several advantages have been mentioned for using the technique in treating long-bones fractures (4). Intramedullary nails make alignment and appropriate rotation possible in treating the fractures. In addition to elasticity and appropriate stability, they result in micromotion at the fractured area, strengthening osseous calculus formation, and finally, acceleration of union process. Small incision is used in surgical treatment and there is very weak probability of infection (8, 9). According to the results of our study, the union time is not different between two methods in children. Major complications in external fixator are more than intramedullary nail. Re-fractures and Leg-length discrepancies are the major complication were observed in our patients who treated with external fixator. There are few studies in the literature on the management of diaphyseal fractures of the tibia in children with intramedullary fixation especially in open fractures. Vallamshetla et al. (4) showed that fixation is an easy and effective method of management of both open and closed unstable fractures of the tibia in children. In this study, the avarege time of union in itramedullay nail was 10 weeks and the major complications were included residual angulation of the tibia, leg-length discrepancy, deep infection and failures of fixation. Unlike this study, such complications were not observed in our patients, but the union time was similar. Deakin et al. (10) study in thirty-five adolescent patients underwent flexible intramedullary nails for tibia was not any nonunion and the union time was higher (17 weeks) than our study. Kubiak et al. (11) recommended when surgical stabilization of tibial fractures in children is indicated, fixation with elastic stable intramedullary nailing is preferred. Griffet et al. (8) study in 86 children with tibial fractures was expressed, the fixation of paediatric diaphyseal tibial fractures with elastic stable intramedullary nailing is a rapid, well-codified and effective method for treating long-bone closed fractures in children.

Advantages over other fixation techniques include a lower infection rate, a lower re-fracture rate, ease of management, and an aesthetically pleasing scar. However, external fixator was associated with quick stability of long-bones fractures. External fixator is one of the effective ways in treating open fractures with severe damage of soft tissues. It lacks some complications of fixator such as infection surrounding pin, need to care, and re-fracture. In our study, combination of pin with flexible intramedullary nails developed maximum stability in severe crush cases. It is a new point considering the previously conducted studies and may be helpful in appropriately treating open fractures. TEN nails method was regarded as an effective method comparable with external fixator in treating open fractures. Combination of pin with TEN nails results in more stability of fracture and is not associated with more complications.

Although external fixation in open pediatric fractures and severe injuries is recommended, intramedullary nailing is also an effective method with low complications. Combining pins and flexible intramedullar nails is effective in developing more stability and is not associated with more complications.

## References

[1] Settera KJ, Palominob KE (2006). Pediatric tibial fractures: current concepts.. Curr Opin Pediatr..

[2] Galano GJ, Vitale MA, Kessler MW, Hyman JE, Vitale MG (2005). The most frequent traumatic orthopaedic injuries from a national pediatric inpatient population.. J Pediatr Orthop..

[3] Mashru RP, Herman MJ, Pizzutillo PD (2005). Tibial shaft fractures in children and adolescents.. J Am Acad Orthop Surg..

[4] Vallamshetla VR, De Silva U, Bache CE, Gibbons PJ (2006). Flexible intramedullary nails for unstable fractures of the tibial in children. An eight-year experience.. J Bone Joint Surg Br..

[5] Pandya NK, Edmonds EW (2012). Immediate intramedullary flexible nailing of open pediatric tibial shaft fractures.. J Pediatr Orthop..

[6] Allison P, Dahan-Oliel N, Jando VT, Yang SS, Hamdy RC (2011). Open fractures of the femur in children: analysis of various treatment methods.. J Child Orthop..

[7] Tolo VT (1983). External skeletal fixation in children's fractures.. J Pediatr Orthop..

[8] Griffet J, Leroux J, Boudjouraf N, Abou-Daher A, El Hayek T (2011). Elastic stable intramedullary nailing of tibial shaft fractures in children.. J Child Orthop..

[9] Ligier JN, Metaizeau JP, Prevot J (1983). [Closed flexible medullary nailing in pediatric traumatology].. Chir Pediatr..

[10] Deakin DE, Winter H, Jain P, Bache CE (2010). Malunion following flexible intramedullary nails for tibial and femoral fractures in adolescents.. J Child Orthop..

[11] Kubiak EN, Egol KA, Scher D, Wasserman B, Feldman D, Koval KJ (2005). Operative treatment of tibial fractures in children: are elastic stable intramedullary nails an improvement over external fixation?. J Bone Joint Surg Am..

